# COVID-19 Concerns, Vaccine Acceptance and Trusted Sources of Information among Patients Cared for in a Safety-Net Health System

**DOI:** 10.3390/vaccines10060928

**Published:** 2022-06-10

**Authors:** Terry C. Davis, Robbie Beyl, Mohammad A. N. Bhuiyan, Adrienne B. Davis, John A. Vanchiere, Michael S. Wolf, Connie L. Arnold

**Affiliations:** 1 Department of Medicine and Feist-Weiller Cancer Center, Louisiana State University Health Shreveport, Shreveport, LA 71103, USA; connie.arnold@lsuhs.edu; 2 Biostatistics & Analysis, Pennington Biomedical Research Center, Louisiana State University, Baton Rouge, LA 70808, USA; robbie.beyl@pbrc.edu; 3 Department of Medicine, Louisiana State University Health Shreveport, Shreveport, LA 71103, USA; nobel.bhuiyan@lsuhs.edu (M.A.N.B.); adrienne.davis@lsuhs.edu (A.B.D.); 4 Department of Pediatrics, Louisiana State University Health Shreveport, Shreveport, LA 71103, USA; john.vanchiere@lsuhs.edu; 5 Division of General Internal Medicine and Geriatrics, Feinberg School of Medicine, Chicago, IL 60611, USA; mswolf@northwestern.edu

**Keywords:** COVID-19 concerns, COVID-19 vaccine acceptance, trusted sources of COVID vaccine information

## Abstract

We examined COVID-19 concerns, vaccine acceptance, and trusted sources of information among patients in a safety-net health system in Louisiana. The participants were surveyed via structured telephone interviews over nine months in 2021. Of 204 adult participants, 65% were female, 52% were Black, 44.6% were White, and 46.5% were rural residents. The mean age was 53 years. The participants viewed COVID-19 as a serious public health threat (8.6 on 10-point scale). Black adults were more likely to perceive the virus as a threat than White adults (9.4 vs. 7.6 *p* < 0.0001), urban residents more than rural (9.0 vs. 8.2 *p* = 0.02), females more than males (8.9 vs. 8.1 *p* = 0.03). The majority (66.7%) had gotten the COVID-19 vaccine, with females being more likely than males (74.7 vs. 54.5% *p* = 0.02). There was no difference by race or rural residence. Overall, participants reported that physicians were the most trusted source of COVID-19 vaccine information (77.6%); followed by the CDC/FDA (50.5%), State Department of Health (41.4%), pharmacists (37.1%), nurses (36.7%); only 3.8% trusted social media. All sources were more trusted among black adults than White adults except family and social media. These findings could help inform efforts to design trustworthy public health messaging and clinical communication about the virus and vaccines.

## 1. Introduction

The COVID-19 pandemic continues to adversely affect public health. In the United States, more than one million individuals have died of the virus, the highest number of deaths of any country in the world [1,2]. Vaccination is an effective approach to preventing infection and reducing mortality due to COVID-19 [3]. Access [1,4], understanding, and trust in authorized vaccines are critical for improving population health [5].

The COVID-19 vaccine first became available to the public in the US on 13 December 2020. As of April 2022, 77.4% of US adults have received at least one dose of the vaccine, and 65.9% were fully vaccinated [3,6]. The Commonwealth Fund estimates that vaccination has saved approximately 2 million lives in the United States alone [7]. Of note, the CDC reports that vaccination against COVID-19 remains uneven across the country [8]. Louisiana currently ranks 48th among states in the percentage of residents who are at least partially vaccinated. Approximately 60% of adults have received at least one dose, and 53% are considered fully vaccinated with at least two doses [9].

The World Health Organization (WHO) considers vaccine hesitancy to be a global health threat [10]. The WHO has defined vaccine hesitancy as behavior influenced by a number of factors, including a lack of trust in the vaccine or the provider, complacency (not perceiving a need for the vaccine or not valuing the vaccine), or a lack of convenient access [10]. Major factors associated with vaccine hesitancy include perceptions of risk, safety, efficacy, trust as well as social demographic characteristics [2,11,12]. Numerous recent studies have found vaccine-hesitant individuals are a heterogeneous group who have varying degrees of indecision about specific vaccines or vaccination in general. Studies also found that COVID-19 vaccine acceptance and behavior change over time.

We conducted a telephone survey among patients with a recent physician visit in a north Louisiana safety-net health system over a nine-month period. The survey focused on COVID-19 vaccines and included a wide age range of adults, especially those experiencing high rates of health disparities, such as low-income individuals, Black adults, and individuals living in rural areas. The purpose of this project was to determine COVID-19-related concerns, COVID-19 vaccine acceptance, and trusted sources of vaccine information. The goal was to aid in the development of effective strategies to enhance public health and clinical communication about the virus and vaccines.

## 2. Materials and Methods

This project took place from February to October 2021. Trained public health students and clinical research assistants called adult patients of Ochsner-LSU Health in Shreveport who had a recent provider visit and asked if they would be willing to participate in a brief telephone survey about COVID-19. The structured survey took approximately 10 min to complete. Participant responses were entered into a REDcap web-based survey database. LSU Health serves low-income patients predominately; 75% have Medicaid and or Medicare, and 12% have private insurance. The project was approved by the LSU Health Shreveport IRB. Participants were not paid for their time.

### 2.1. Participants

All of the participants were ambulatory care patients at least 18 years of age who had participated in at least one visit with a provider at a non-urgent care clinic during the study period.

### 2.2. Measurement

The 6-item survey was designed to assess participants’ COVID-19 concerns as well as COVID-19 vaccine experience, concerns, and trusted sources of information. The survey was modified from one developed by Wolf and colleagues [13,14]. Participant characteristics elicited included age, race, sex, employment status, and location of residence (city or small town/rural).

### 2.3. COVID-19 Concerns

Concern was assessed by asking, “How serious a public health threat do you think coronavirus is or might become?” This was scored on a scale of 1–10, with 10 being a very serious threat.

### 2.4. COVID-19 Vaccine Experience and Concerns

COVID-19 vaccine experience was assessed with four questions asking if they had gotten the vaccine, and, if so, were they given any information about side effects and if their doctor had talked with them about receiving the vaccine. Answers were coded as yes or no. Vaccine concerns were assessed by asking those who were not planning to take the vaccine, “why” with a list of 12 options from which they could select all that applied (allergic to the vaccine, concerned about becoming really sick, concerned about vaccine side effect(s), against my religion, want to wait, etc.).

### 2.5. Trust in COVID-19 Information

One item assessed trusted sources of COVID-19 vaccination. Ten options were listed from which they could choose all that applied. Answers included: the President, the CDC, the Louisiana State Department of Health, your doctor, nurse, pharmacist, minister, family, friends, and social media.

### 2.6. Statistical Analysis

For analysis purposes, demographic characteristics groups were defined as follows: sex (male and female), race (Black, White), age (18–29, 30–44, 45–64, and 65+), location (urban and rural), and employment status (working for pay, not working/retired). T-tests were used to test group differences on the Likert scale scores after checking the residuals for departures from normality. Chi-Square tests were used to determine statistically significant differences between groups and categorical responses. The results are given as means and standard deviations or percentages. All of the available data were used in the analysis, and, thus, each characteristic group has its own total sample size.

## 3. Results

Of the 378 participants in a specialty or primary care clinic in one health system contacted by phone, 204 completed the survey with a response rate of 54.0%. Table 1 summarizes the characteristics of participants; the mean age was 53 years, the majority were female (65.2%), 52.0% were Black, 44.6% were white, and 46.5% reported living in rural areas. A total of 77.8% reported not working for pay.

### 3.1. COVID-19 Concerns

The participants viewed COVID-19 as a serious public health threat; 8.6 on a scale of 1–10. Black adults reported being more concerned than White adults (9.4 vs. 7.6, *p* < 0.0001), and females were more concerned than males (8.9 vs. 8.1, *p* = 0.03). Urban residents were more concerned than rural residents (9.0 vs. 8.2, *p* = 0.02).

### 3.2. COVID-19 Vaccine Experience and Concerns

Overall, the majority (83.3%) reported their doctor had talked to them about receiving a COVID-19 vaccine, yet fewer (66.4%) had received at least one dose of the COVID-19 vaccine. There was no difference by race, sex, or rural residence in talking to the doctor about COVID vaccines. Females were more likely to have reported receiving a COVID-19 vaccine than males (74.7% vs. 54.5%, *p* = 0.02); there was no difference by race or rural residence.

Of those that had received a COVID-19 vaccine, 76.7% reported having been given information on side effects. Participants living in rural areas were more likely to have received side effect information (86.0% vs. 68.2%, *p* = 0.047) compared to those living in urban areas.

The most common reasons participants reported for not planning to recieve a COVID-19 vaccine were concern about side effects (56.1%), they do not trust that the vaccine will be safe (32.9%), and they do not think that the vaccine works very well (14.6%). There was no difference in the responses by race, sex, or rural residence (Table 2).

### 3.3. Trusted Sources for COVID-19 Vaccine Information

Physicians were the most trusted (79.7%) source of COVID-19 vaccine information, followed by federal agencies (CDC/FDA) (52.0%) and the Louisiana State Department of Health (42.2%) (Table 3). More than one-third of respondents reported trusting pharmacists (38.2%) or nurses (37.7%). Less than one-fourth of respondents trusted local newspapers or TV (25.0%), their minister (24.0%), the President (23.5), or their family (22.5%). The participants reported Facebook/social media as the least trusted (3.9%). All sources of COVID-19 vaccine information were more trusted among Black adults than White adults except for family and social media. Females were more likely to report trusting their doctors (84.1% vs. 72.2%, *p*= 0.043) and federal health agencies (60.6% vs. 36.1%, *p* = 0.001) than males. There was no difference in trust among those that lived in urban areas vs. rural.

## 4. Discussion

In a COVID-19 vaccine-related telephone interview with predominantly low-income participants in a Southern safety-net health system, the majority of participants perceived the SARS-CoV-2 virus as a serious public health threat. All had had a physician visit during the study period, and most said their physician had talked to them about COVID-19 vaccines. However, approximately one in three in this 2021 survey reported they did not intend to take the vaccine. Males were less likely to intend to take the vaccine than females, but there was no difference by race or urban vs. rural residence.

Unlike our findings, Khubchandani and colleagues, in a comprehensive review of 13 national studies, found that vaccine hesitancy was higher among Black adults and rural residents [15]. In a community-based survey in 2021 of predominately Black adults in Georgia, Moore and colleagues found that nearly one in three people were hesitant to take a COVID-19 vaccine [16]. The same study revealed that younger participants (under age 30) and those experiencing housing insecurity because of the pandemic were more likely to be hesitant to receive the vaccine [16]. The same study revealed that younger participants (under age 30) and those experiencing housing insecurity because of the pandemic were more likely to be resistant to receiving the vaccine [16]. The fact that we did not find more vaccine hesitancy among Blacks in our study may be due to several reasons: (1) The Black community in our area experienced significantly greater COVID-19 hospitalization and death, and this was widely publicized in the local media; (2) the Louisiana Department of Health and our Health System partnered with Black civic and faith-based groups to conduct ongoing community outreach efforts to provide vaccines that were easily accessible and messaging that was culturally appropriate, easy to understand and acceptable to Black adults in our area.

Previous studies have found that the intention to be vaccinated was related to the perception of the virus being a threat and perceived personal risk, as well as the safety, efficacy, and trust in the vaccine [2,10,11]. In our study, most participants perceived the virus to be a threat. The main reasons for not being vaccinated were concerns about vaccine safety and side effects. Participants living in cities were more concerned with vaccine efficacy and side effects than those living in rural areas.

As revealed by others, the most trusted sources of information regarding COIVD-19 vaccines were health care providers and federal and state health agencies, moderate levels of trust were found in the media and lower levels of trust in social media [17,18,19]. In an online study of Arkansas residents in 2020, the participants’ main reason for lack of trust in information was that the information was rapidly changing information and there was a lack of consistency across sources. Participants found changing and contradictory information confusing [19]. In our study, trust in all sources of information increased over time, with doctors, pharmacists, and state and federal health agencies being the most highly trusted; social media and Facebook rated the lowest source of trusted information, and Black adults were more trusting than White adults. This differs from an earlier national survey that found trust in information was rapidly decreasing, and trust in federal, state, and local governments was declining [20]. Our findings do not indicate why trust increased, but it may be in part because of the targeted community outreach in which the health systems partnered with trusted community groups. Consistent, concise, and trustworthy media messaging by local healthcare leaders working in partnership with local civic, public health, and government leaders has been a notable strength of the COVID-19 response efforts in our region.

Given the unpredictable future of COVID-19, the emergence of new variants, and potentially waning population immunity, there will be a continuing need for clear, trustworthy public health and clinical communication [8]. Health information for the public needs to be conveyed in plain language and be easy to understand to help individuals make informed health decisions [21].

### Limitations

Several limitations of this study are important: (1) It was a relatively small convenience sample with predominantly low-income patients at one health system; (2) The survey was conducted in English only; and (3) vaccination information and utilization of various information sources were not verified or corroborated for each participant.

## 5. Conclusions

This study provides a snapshot of low-income Louisiana patients’ COVID-19 concerns, vaccine acceptance, and trusted sources of information. The findings are important to help inform ongoing efforts to increase effective clinical and public health communication about vaccines to promote trust. Additionally, the research points to the importance of regional studies to help inform public health messaging and outreach strategies and the need to encourage providers to continue to communicate trustworthy information about vaccine safety and efficacy in their communication with patients. Providers and public health professionals should consider emphasizing vaccine efficacy and targeting strategies to male patients. Disparities in public perceptions and uptake of COVID-19 vaccines continue to evolve, and research over time is needed on vaccine acceptance among a wide range of adults and their providers to understand the complex factors that promote and mitigate vaccine hesitancy. As the pandemic continues, variant COVID-19 strains emerge, and vaccine efficacy wanes, ongoing research is needed that addresses the changing perceptions of infection/disease severity, risks and benefits of vaccination and boosters, and issues related to children and families.

## Figures and Tables

**Table 1:** Demographic Characteristics of Participants (*n* = 204).

Characteristic	*n*
**Total**	204
**Age, mean (SD)**	53.2 (13.9)
	*n* (%)
**Age**	
18–29	11 (5.4)
30–44	49 (24.0)
45–64	104 (51.0)
65+	40 (19.6)
**Sex**	
Female	129 (65.2)
Male	71 (34.8)
**Race**	
Black or African American	106 (52.0)
White	91 (44.6)
Other	7 (3.4)
**City of Residence**	
Urban	106 (53.5)
Rural	92 (46.5)
**Current Employment Situation**	
Working now for pay	43 (22.2)
Not working for pay	151 (77.8)

**Table 2:** COVID-19 Vaccine Experiences and Concerns by Race, Sex, and Place of Residence.

	Race			Sex			Urban vs. Rural		
**Experiences and Concerns**	**Black**	**White/Other**		**Female**	**Male**		**Urban**	**Rural**	
**Totals**	% (*n*/total)	% (*n*/total)	*p*-value	% (*n*/total)	% (*n*/total)	*p*-value	% (*n*/total)	% (*n*/total)	*p*-value
**Doctor discussed recieving COVID-19 vaccine**	86.2 (56/65)	80.8 (42/52)	0.433	86.8 (66/76)	77.3 (34/44)	0.175	82.1 (46/56)	84.6 (55/65)	0.715
**Recieved a COVID-19 vaccine**	61.5 (40/65)	71.2 (37/52)	0.213	74.7 (56/75)	54.5 (24/44)	0.02 *	69.1 (38/55)	64.6 (42/65)	0.604
**Reasons for not recieving/or planning to take vaccine:**									
Concerned about side effects from vaccine	65.0 (26/40)	47.6 (20/42)	0.113	61.2 (30/49)	50.0 (16/32)	0.319	61.4 (27/44)	51.4 19/37)	0.348
Don’t think vaccine works very well	10.0 (4/40)	19.0 (8/42)	0.247	14.3 (7/49)	15.6 (5/32)	0.904	9.1 (4/44)	21.6 (8/37)	0.259
Don’t trust that the vaccine is safe	30.0 (12/40)	35.7 (15/42)	0.582	34.7 (17/49)	31.3 (10/32)	0.740	27.3 (12/44)	40.5 (15/37)	0.350
**Provided information about side effects**	75.6 (34/45)	77.5 (31/40)	0.833	76.3 (45/59)	77.8 (21/27)	0.878	68.2 (30/44)	86.0 (37/43)	0.047 *

* Significant at the *p* < 0.05 level.

**Table 3:** Trusted Sources for COVID-19 Vaccine.

	Race			Sex			Urban vs. Rural		
	**Black**	**White**		**Female**	**Male**		**Urban**	**Rural**	
	*n* = 108	*n* = 91	*p*-value	*n* = 132	*n* = 72	*p*-value	*n* = 107	*n* = 93	*p*-value
	% (*n*)	% (*n*)		% (*n*)	% (*n*)		% (*n*)	% (*n*)	
**Sources**									
The President	32.4 (35)	11.0 (10)	0.0003 **	26.5 (35)	18.1 (13)	0.173	20.6 (22)	28.0 (26)	0.222
Health agencies (CDC, FDA, etc.)	59.3 (64)	42.9 (39)	0.021 *	60.6 (80)	36.1 (26)	0.001 **	53.3 (57)	52.7 (49)	0.934
LA State Dept of Health	51.9 (56)	30.8 (28)	0.003 *	45.5 (60)	36.1 (26)	0.197	45.8 (49)	40.9 (38)	0.483
LA state government	34.3 (37)	17.6 (16)	0.008 *	28.8 (38)	25.0 (18)	0.562	25.2 (27)	31.2 (29)	0.350
Local News stations or newspapers	29.6 (32)	17.6 (16)	0.048 *	23.5 (31)	27.8 (20)	0.499	22.4 (24)	29.0 (27)	0.285
Doctor/health care provider	84.3 (91)	72.5 (66)	0.043 *	84.1 (111)	72.2 (52)	0.043 *	81.3 (87)	81.7 (76)	0.940
Nurse	44.4 (48)	28.6 (26)	0.021 *	37.9 (50)	37.5 (27)	0.958	38.3 (41)	38.7 (36)	0.955
Pharmacist	43.5 (47)	30.8 (28)	0.065	36.4 (48)	41.7 (30)	0.456	35.5 (38)	43.0 (40)	0.278
Minister/faith leader	30.6 (33)	16.5 (15)	0.021 *	22.0 (29)	27.8 (20)	0.353	25.2 (27)	23.7 (22)	0.796
Your family	25.9 (28)	16.5 (15)	0.107	22.0 (29)	23.6 (17)	0.789	17.8 (19)	29.0 (27)	0.059
Facebook or social media	(6)	(2)	0.230	(3)	(5)	0.100	(3)	(5)	0.354

* Significant at the *p* < 0.05 level. ** Significant at the *p* < 0.001 level.

## Data Availability

The data presented in this study are available on request from the corresponding author. The data are not publicly available due to the protection of personal health information of participants.
